# The nervous system of *Paludicella articulata* - first evidence of a neuroepithelium in a ctenostome ectoproct

**DOI:** 10.1186/s12983-014-0089-2

**Published:** 2014-12-10

**Authors:** Anna V Weber, Andreas Wanninger, Thomas F Schwaha

**Affiliations:** Department of Integrative Zoology, University of Vienna, Althanstraße 14, 1090 Vienna, Austria

## Abstract

**Introduction:**

Comparatively few data are available concerning the structure of the adult nervous system in the Ectoprocta or Bryozoa. In contrast to all other ectoprocts, the cerebral ganglion of phylactolaemates contains a central fluid-filled lumen surrounded by a neuroepithelium. Preliminary observations have shown a small lumen within the cerebral ganglion of the ctenostome *Paludicella articulata*. Ctenostome-grade ectoprocts are of phylogenetic relevance since they are considered to have retained ancestral ectoproct features. Therefore, the ctenostome *Paludicella articulata* was analyzed in order to contribute to the basal neural bauplan of ctenostomes and the Ectoprocta in general.

**Results:**

The presence of a lumen and a neuroepithelial organization of the nerve cells within the cerebral ganglion are confirmed. Four tentacle nerves project from the cerebral ganglion into each tentacle. Three of the tentacle nerves (one abfrontal and two latero-frontal nerves) have an intertentacular origin, whereas the medio-frontal nerve arises from the cerebral ganglion. Six to eight visceral nerves and four tentacle sheath nerves are found to emanate from the cerebral ganglion and innervate the digestive tract and the tentacle sheath, respectively.

**Conclusions:**

The situation in *P. articulata* corresponds to the situation found in other ctenostomes and supports the notion that four tentacle nerves are the ancestral configuration in Ectoprocta and not six as proposed earlier. The presence of a lumen in the ganglion represents the ancestral state in Ectoprocta which disappears during ontogeny in all except in adult Phylactolaemata and *P. articulata*. It appears likely that it has been overlooked in earlier studies owing to its small size.

## Introduction

Bryozoa or Ectoprocta are widespread colonial suspension-feeders and predominantly marine animals mainly attached to hard substrates. They represent a large lophotrochozoan phylum with over 6.000 recent and about 15.000 extinct species. In various phylogenetic analyses ectoprocts were placed at different positions within the Bilateria [1-3]. Owing to several morphological similarities of the adults, in particular the lophophore, ectoprocts were traditionally united with phoronids and brachiopods in the clade Lophophorata [4], which also receives support in some recent molecular analyses [5,6]. Monophyly of Brachiopoda and Phoronida is often found in molecular phylogenies, but to the exclusion of Ectoprocta [1,2,7-9].

Within Ectoprocta, three major subtaxa are commonly recognized: Phylactolaemata, Stenolaemata and Gymnolaemata, the latter comprising the Ctenostomata and Cheilostomata. An ectoproct colony (zooarium) is composed of single individuals called zooids. Each zooid has a tough, often calcified reinforced body wall, the cystid, in which the soft part, named polypide, may retract into. The polypide mainly consists of the lophophore and digestive tract and is used for food uptake [4,10].

Phylactolaemata is often regarded as the sister-taxon to all remaining Ectoprocta. However, the relationships within the ectoproct subtaxa remain controversial. The “Ctenostomata” are currently regarded as paraphyletic [11,12]. They are little investigated and can be divided into two subclades, Carnosa and Stolonifera [4]. Ctenostomes lack a mineralized cystid and, thus, complex calcified structures. Concerning bryozoan soft body morphology only little data and mostly old monographs are available [13-16] with only a small amount of recent studies focusing on specific organ systems [17-20]. As a consequence, there is limited data available concerning the adult ectoproct nervous system. Some of the earliest notes on the nervous system are available in old monographs (e.g. [14]). Only a few studies specifically addressing the nervous system were conducted in the early 20th century (e.g. [21,22]). The most profound knowledge on the nervous system was gained by a series of studies of Lutaud (e.g. [23-26]), also summarized in Mukai et al. [4]. In the last decades only a few notable studies had a focus on adult nervous systems (e.g. [20,27]).

In adult Phylactolaemata the serotonergic nervous system is concentrated in the cerebral ganglion, from which a serotonergic neurite extends to each tentacle base [28]. Investigations showed that the cerebral ganglion of adult Phylactolaemata bears a small fluid-filled lumen [14]. An organization of the nervous cells as a neuroepithelium that bears interconnections of neurons via adherens junctions was described [27]. The ontogenetic origin of the cerebral ganglion has been described as an invagination of the inner layer of the bilayered bud, i.e. ultimately derived from the epidermis of the mother zooid, in all bryozoan taxa investigated so far. Consequently, in the ganglion of early developmental stages, there is a lumen which is described to disappear during development in all clades except in the Phylactolaemata [20,29,30].

Taken together, the data currently available show that the adult ectoproct nervous system is rather simple and mainly consists of a cerebral ganglion at the base of the lophophore, a circum-oral/circum-pharyngeal nerve ring and nerves emerging from the cerebral ganglion - which constitute the tentacular and tentacle sheath innervation - as well as some nerve fibers that project to the gut. Simultaneous response to environmental cues of several zooids (or heterozooids) raise questions concerning neural communication between individual zooids within a colony. In the cheilostome *Electra pilosa* [25] as well as in other malacostegine cheilstome ectoprocts [31] a chain of perikarya at the base of the longitudinal and transverse parts of the cystid was described. These perikarya are associated with parietal filaments and are linked along the tentacle sheath with the cerebral ganglion, and the filaments of neighboring zooids are periodically connected through pore plates [24]. In the cheilostome *Electra pilosa* and the ctenostome *Hislopia malayensis* 4 basiepidermal tentacle nerves were described [23,32]. In the cheilostome *Cryptosula pallasiana* two additional basiperitoneal tentacle nerves are present [18]. In the Phylactolaemata all tentacle nerves have an intertentacular origin and form intertentacular forks [21]. Conversely, in Gymnolaemata tentacle nerves branch off directly from the cerebral ganglion/nerve ring. In the ctenostome *H. malayensis* the medio-frontal nerve emerges directly from the cerebral ganglion, whereas the abfrontal and the latero-frontal nerves emanate from intertentacular forks [32]. In the cheilostomate *E. pilosa* the medio-frontal as well as the abfrontal nerve branches off directly from the cerebral ganglion [23]. This trend reflects the current phylogenetic view that the Phylactolaemata represent a basal ectoproct offshoot and that the Ctenostomata are paraphyletic [11,12,32,33].

In the current study special focus was on the innervation of the tentacles in order to assess whether the tentacle nerves show an intertentacular origin or whether they emanate directly from the cerebral ganglion. Preliminary data also showed a lumen within the cerebral ganglion of *Paludicella articulata* (Ctenostomata) (T. Schwaha, pers. observation). This has never been described in the Ctenostomata before. In order to test for a neuroepithelial organization of the nervous cells within the cerebral ganglion (see [27]), to analyze the hollow structure of the cerebral ganglion in more detail, and to gain more insight into the neural anatomy of *P. articulata*, the zooids were studied at histological and ultrastructural level. In addition, confocal microscopy analyses of specimens stained for the pan-neural marker α-tubulin were performed.

## Materials and methods

### Fixation, staining and image acquisition

Adult colonies of *Paludicella articulata* were sampled at the Laxenburg pond, Lower Austria, at a depth of 0.1-0.5 m and were transferred to the laboratory. Some of the zooids were anesthetized by adding few drops of chloral hydrate and subsequently fixed either for immunocytochemical staining, transmission electron microscopy or light microscopy. For comparison, some specimens were fixed with retracted polypides (without chloral hydrate for relaxation). Fixation for immunocytochemistry was done in 4% paraformaldehyde (PFA) in 0.1 M phosphate buffer (PB) with 0.01% NaN_3_ for 1 hour at room temperature. Subsequently, samples were rinsed three times for 20 minutes and stored in PB with 0.01% NaN_3_ added. To permeabilize the tissues, colony branches were dissected and washed three times for 10 minutes in PB to remove the NaN_3_. Next, the samples were blocked overnight at room temperature in 6% normal goat serum in PB with 4% Triton X-100 (Sigma Aldrich, St. Louis, MO, USA). A monoclonal mouse anti-acetylated α tubulin primary antibody (ImmunoStar, Hudson, USA) was applied in a concentration of 1:500 blocking solution in PB. In the following, the specimens were rinsed in the blocking solution three times for two hours at room temperature. Then, the secondary goat anti-mouse antibody conjugated to the fluorescent dye Alexa Fluor 488 (Invitrogen, Carlsbad, CA, USA) was added in a 1:500 dilution to the blocking solution. Nuclear counterstaining was performed with 4′6-diamidino-2-phenylindole (DAPI, Sigma, Buchs SG, Switzerland) in a concentration of 1:300. The specimens were incubated overnight and finally washed three times for a total of 1.5 hours in PB without NaN_3_. All samples were embedded either in Fluoromount G (Southern Biotech, Birmingham, AL, USA) or Vectashield (Vector Laboratories, Burlingame, CA, USA) on glass slides.

Stained samples were scanned with a Leica TCS SP5 II (Leica Microsystems, Wetzlar, Germany) confocal laser scanning microscope (CLSM) with a step size of 0.5 μm-1 μm along the Z-axis. Image stacks were merged into maximum projections or rendered three-dimensionally with the aid of the reconstruction software Amira 5.4 software (FEI Visualization Science Group, Hillsborow, OR, USA).

### Transmission electron microscopy, light microscopy and 3D reconstruction

Zooids or colony parts were prefixed according to Gruhl and Bartolomaeus [27] in a 2.5% glutaraldehyde solution in 0.01 M PB (pH 7.4) at room temperature for one hour. Specimens were then rinsed three times for 20 minutes in PB. For postfixation, the samples were treated with 1% osmium tetroxide in PB for 1 hour at room temperature. Dehydration was done via acidified dimethoxypropane and afterwards embedded in Agar low-viscosity resin (LVR, Agar Scientific, Stanstead, Essex, UK) using acetone as intermediate. After overnight polymerization at 60°C, semi-thin (1 μm thickness) and ultrathin (60 nm thickness) serial sections were generated with Diatome diamond knifes (Diatome, Biel, Switzerland) on a Leica UC6 ultramicrotome (Leica Microsystems, Wetzlar, Germany). Ultra-thin sections were collected on mesh copper grids or on formvar-coated single-slot copper grids. Staining was carried out with uranylacetate for 35–40 minutes and lead citrate for 5 minutes. Ultrathin sections were examined on a Zeiss Libra 120 and a Zeiss EM 902 transmission electron microscope (Carl Zeiss AG, Oberkochen, Germany). Images were adjusted for brightness and contrast using Adobe Photoshop CS5 (Adobe, San Jose, CA, USA).

Semi-thin sections were mounted in LVR after staining with toluidine blue for 5–10 seconds at 60°C. Photographs were taken with a Nikon Eclipse E800 light microscope (Nikon, Chiyoda, Tokio, Japan) equipped with a Nikon Fi2-U3 microscope camera. Images were transformed into grayscale format with Adobe Photoshop CS5 and then imported into the reconstruction and visualization software Amira 5.4. Alignment of the image stack was conducted with the Align Slices tool. Structures of interest (tentacle nerves, cerebral ganglion, peritoneum, epidermis) were manually segmented. Based on the segmentation data, surfaces were generated and were optimized via successive alternating steps of polygon-reduction and additional surface smoothing. Snapshots of the 3D models were taken with the Amira software (see also [34]).

## Results

### Gross morphology of *Paludicella articulata*

**Figure 1:**
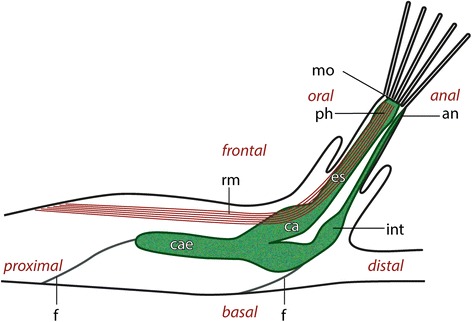
**Schematic representation of a lateral view through a zooid of** ***Paludicella articulata*** **after Allman [** 36 **].** Abbreviations: an, anus; ca, cardia; cae, cecum; es, esophagus; f, funiculus; int, intestine; mo, mouth opening; rm, retractor muscle; ph, pharynx.

**Figure 2:**
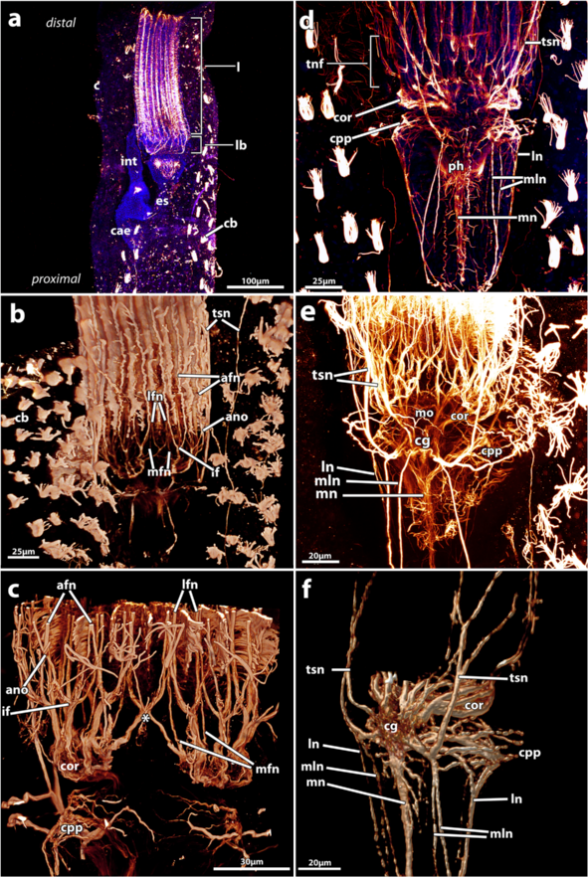
**Adult** ***Paludicella articulata*** **.** Maximum projections of confocal image stacks. Fluorescence staining with antibodies against acetylated α-tubulin (red) and in part staining of nucleic acids by DAPI (blue). **a)** overlay of acetylated α-tubulin and DAPI signal for an overview of the nervous system. **b)** overview of the central nervous system with focus on the tentacle innervation; volume rendering based on confocal image stack. **c)** Detail of the tentacle innervation by 3 different nerve types; medio-frontal tentacle nerves arise directly from the cerebral ganglion; the abfrontal and the paired latero-frontal tentacle nerves emerge from a intertentacular origin (intertentacular fork and abfrontal nerve origin); volume rendering based on confocal image stack. **d)** Overview of the acetylated α-tubulin-immunoreactive elements of the central nervous system and from the nerve strands that emerge from the cerebral ganglion. **e)** Detailed view of all nerves that arise from the cerebral ganglion: visceral nerves, tentacle sheath nerves, and tentacle innervation. **f)** Oblique view of the visceral nerves and tentacle sheath nerves; volume rendering based on confocal image stack. Abbreviations: ano, abfrontal nerve origin; afn, abfrontal nerve; c, cystid; ca, cardia; cae, cecum; cb, ciliary bundle; cg, cerebral ganglion; cor, circum-oral nerve ring; cpp, circum-pharyngeal plexus; es, esophagus; if, intertentacular forks; int, intestine; l, lophophore; lb, lophophore base; ln, latero visceral nerve; lfn, latero-frontal nerve; mfn, medio-frontal nerve; mln, medio-lateral visceral nerve; mn, medio visceral nerve; mo, mouth opening; ph, pharynx; tnf, area of tentacle nerve forks; ts, tentacle sheath; tsn, tentacle sheath nerve; asterisks, abanal closing of the circum-oral nerve ring via fine neurites.

**Figure 3:**
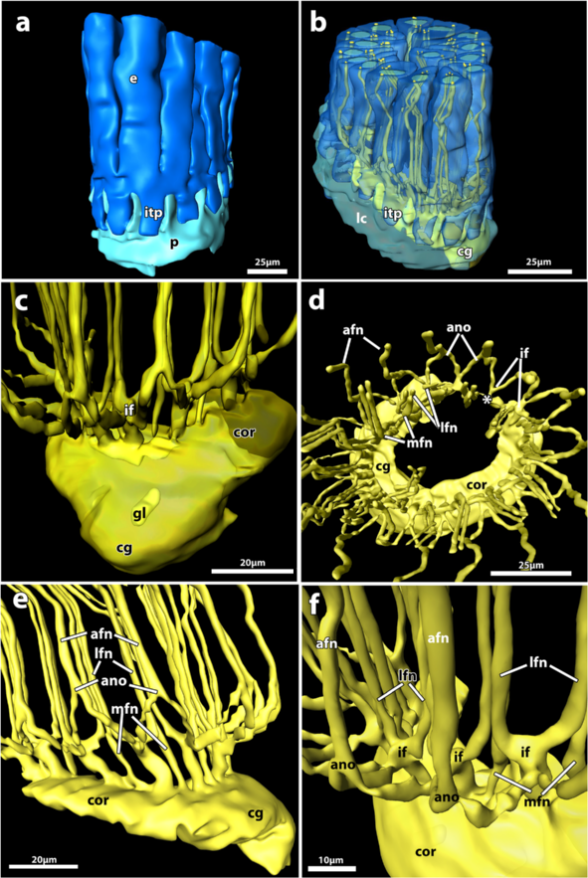
**3D-reconstruction based on semi-thin cross-section series of an adult zooid of** ***Paludicella articulata*** **. a)** Lateral view of the base of the lophophore, showing the epidermis and the peritoneum. **b)** Lateral view, epidermis and peritoneum displayed transparently, tentacle innervation visible below the epidermis. **c)** Lateral view of the cerebral ganglion, parts of the circum-oral nerve ring and the tentacle nerves from the oral side, nervous structures displayed transparently to allow for visibility of the lumen within the cerebral ganglion. **d)** Distal view of the central nervous system, showing the circum-oral nerve ring, the tentacle nerves and the cerebral ganglion. **e)** Slightly oblique lateral view of the circum-oral nerve ring, the tentacle innervation and the cerebral ganglion on the anal side to demonstrate the increase of nervous mass towards the anal side. **f)** Oblique view of the tentacle nerves, showing the direct origin of the medio-frontal tentacle nerve and the intertentacular origin of the other tentacle nerves as intertentacular forks and abfrontal nerve origins. Abbreviations: ano, abfrontal nerve origin; afn, abfrontal nerve; cg, cerebral ganglion; cor, circum-oral nerve ring; e, epidermis; gl, ganglion lumen; if, intertentacular forks; itp, intertentacular pit; lc, lophophore coelom; lfn, latero-frontal nerve; mfn, medio-frontal nerve; p, peritoneum; asterisks, abanal closing of the circum-oral nerve ring via fine neuritis Colors: dark blue, epidermal layer of the lophophoral base; light blue, peritoneum; yellow, nervous system.

**Figure 4:**
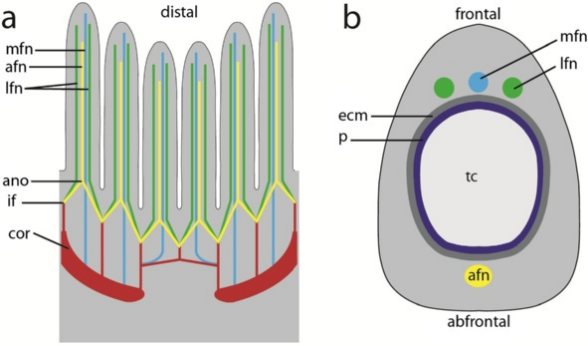
**Schematic representations of the tentacle innervation and the central nervous system of** ***Paludicella articulata*** **. a)** Sagittal view of the lophophore and the lophophoral base. **b)** Cross-section through one tentacle of the lophophore, demonstrating the tentacle innervations by 4 tentacle nerves. Abbreviations: ano, abfrontal nerve origin; afn, abfrontal tentacle nerve; cor, circum-oral nerve ring; ecm, extracellular matrix; if, intertentacular fork; lfn, latero-frontal tentacle nerve; mfn, medio-frontal tentacle nerve; p, peritoneum; tc, tentacle coelom.

**Figure 5:**
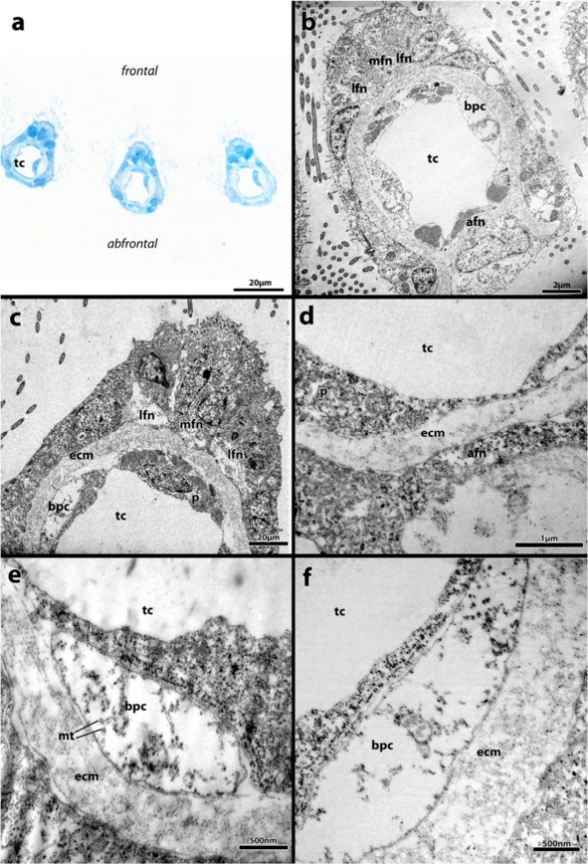
**Cross-sections of tentacles of** ***Paludicella articulata*** **. a)** Semi-thin section through 3 tentacles. **b)** TEM-Overview of a tentacle cross-section, with the 4 tentacle nerves, the 2 basiperitoneal cells and the centrally situated tentacle coelom visible. **c)** Detail of the 3 frontal tentacle nerves. **d)** Detail of the ultrastructure of an abfrontal tentacle nerve. **e)** Detailed view of a basiperitoneal cells with inherent sections of microtubules. **f)** TEM-Image of a basiperitoneal cell, also with sections through inherent microtubules. Abbreviations: afn, abfrontal tentacle nerve; bpc, basiperitoneal cell; ecm, extracellular matrix; lfn, latero-frontal tentacle nerve; mfn, medio-frontal tentacle nerve; mt – microtubules, p, peritoneum; spc – subperitoneal cell; tc, tentacle coelom.

*Paludicella articulata* forms very simple thread-like or runner-like colonies on various substrates in quiet freshwater streams. Within the colony the individual zooids are arranged one after another and form branches [35]. Colonies of *P. articulata* consist of sometimes creeping but more often elongated, mostly erect, slender zooids. There are always 3 adjacent zooids: one distally and two lateral ones. The cuticle (ectocyst) is uncalcified and chitinous. The cystid essentially is the protective body-wall into which the entire polypide may retract if disturbed. The polypide mainly consists of the digestive tract and the lophophore which carries the tentacles. The digestive tract can be divided into the pharynx, esophagus, cardia, cecum, and intestine (Figure 1; Figure 2a). In the current investigation intertentacular pits were found between adjacent tentacles of the lophophore in all semi- and ultrathin sections as a proximal indentation of the tentacle epidermis. The average depth of the intertentacular pits is about 20 μm and the diameter is 7 μm (Figure 3a). Between the epidermal layer of the tentacles and the peritoneum a prominent extracellular matrix is situated (Figure 4b, Figure 5b-d). The tentacle coeloms extend from the lophophoral ring coelom at the lophophoral base into each tentacle.

### Gross morphology of the cerebral ganglion

The most complex part of the polypide is the lophophoral base, where also the main components of the nervous system are situated. The highest concentration of nervous cells is found in the cerebral ganglion from where the circum-oral nerve ring and a circum-pharyngeal nerve plexus emerge. The circum-oral nerve ring surrounds the mouth opening and thus the most distal part of the pharynx. This nerve ring is closed on the side opposite the ganglion by only a few nerve strands. As a result there is a considerable increase in the concentration of perikarya within the circum-oral nerve ring towards the ganglion. The circum-pharyngeal nerve plexus surrounds the pharynx proximal to the circum-oral nerve ring. It mainly consists of 6 nerve strands that project orally. Three nerves emerge on each side of the cerebral ganglion. In contrast to the circum-oral nerve ring the circum-pharyngeal plexus is open at the oral side (Figure 2; Figure 3).

**Figure 6:**
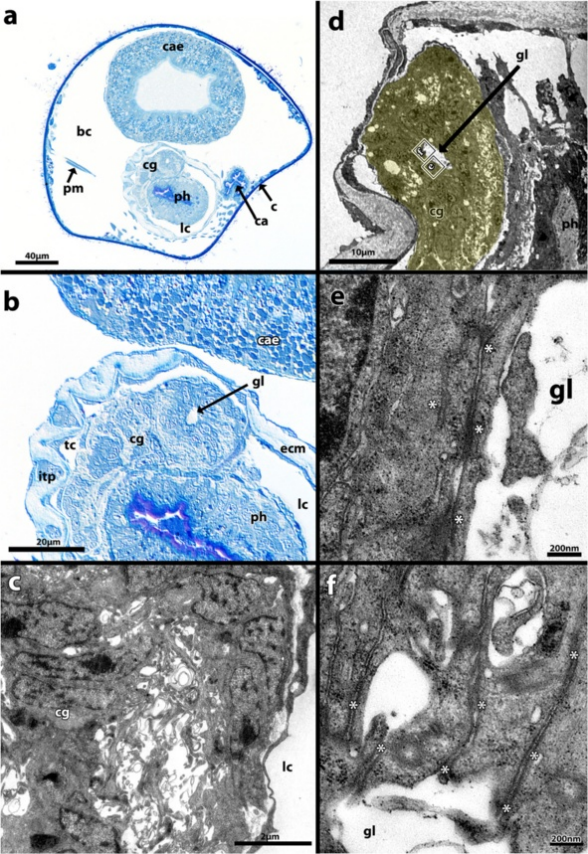
**Semi-thin (a, b) and ultra-thin sections (c-f) of the cerebral ganglion of** ***Paludicella articulata*** **. a)** Semi-thin cross section through the entire zooid, demonstrating the situation of the cerebral ganglion within the zooid **b)** Detailed view of the ganglion lumen in a retracted situation. **c)** TEM micrographs of the ganglionic tissue. **d)** Overview of the cerebral ganglion in a retracted polypide with its small lumen. **e)** Cell connections via adherens junctions in the area of the lumen within the cerebral ganglion. **f)** Adherens junctions of a nerve cell adjoining the fluid-filled lumen. Abbreviations: bc, body coelom; c, cystid; ca, cardia; cae, cecum; cg, cerebral ganglion; ecm, extracellular matrix; gl, ganglion lumen; itp, intertentacular pit; lc, lophophore coelom; ph, pharynx; pm, parietal muscle; tc, tentacle coelom; asterisks, cell-cell connections via adherens junctions.

As mentioned above the majority of nerve cells can be found in the cerebral ganglion, which is located on the anal side between the lophophoral base and the first esophageal subdivision of the digestive tract at the level of the pharynx (Figure 6a, b). It is completely enveloped by an extracellular matrix which clearly separates it from the pharyngeal epithelium. Analyses of semi- and ultrathin sections indicate that the cerebral ganglion contains a central fluid-filled lumen (Figure 6b, d-f).

### Fine structure of the cerebral ganglion

The cerebral ganglion contains neurites, with characteristic neurotubules, and numerous perikarya. The latter are centrally arranged, surrounding and facing the lumen. All cells within the cerebral ganglion show a similar subcellular constitution, i.e. no difference between the ganglionic cells concerning the distribution and abundance of their cell organelles was recognizable. All nerve cells were of the same size and no difference in staining (light microscope) and contrast (TEM) appeared (Figure 6c). TEM investigations showed interconnections of ganglionic cells by adherens junctions in the area surrounding the lumen within the cerebral ganglion (Figure 6e, f). This finding is typical for an organization of the nervous tissue as a neuroepithelium.

### Nerves projecting from the cerebral ganglion

#### Tentacle innervation

The lophophore is innervated by perioral nerve tracts. The gut and the tentacle sheath are innervated by further nerves branching off the cerebral ganglion. Each tentacle is innervated by four basiepidermal nerve fibers, which were identified in all microscopic analyses (CLSM, TEM, light microscopy). Each tentacle possesses one abfrontal nerve, one medio-frontal nerve and one pair of latero-frontal nerves. Three tentacle nerves emerge from an intertentacular fork, located between two adjacent tentacles. The abfrontal nerve arises from the fusion of the two abfrontal nerve roots (Figure 2a-d; Figure 3; Figure 4, Figure 5a-d). Only the medio-frontal nerve branches off directly from the cerebral ganglion/circum-oral nerve ring. Instead of separate nerve strands described in other species (see above), two basiperitoneal cells are present in *P. articulata*. Within these basiperitoneal cells individual microtubules were observed in some ultrathin sections, but these never appeared as distinct as the neurotubules in the four tentacle nerves (Figure 5e, f). No basiperitoneal nerves could be detected in the α-tubulin staining. Thus, it appears likely that the basiperitoneal cells do not constitute nerves.

#### Visceral innervation

In *Paludicella articulata* six to eight visceral nerves branch off the cerebral ganglion (Figure 2d-f). There is one conspicuous median visceral nerve, which follows the esophagus in proximal direction. This nerve strand is the most prominent and always larger than the remaining visceral nerves. The medio-lateral visceral nerves are not bilaterally symmetrical and the number of nerves can differ between zooids. They appear as three to five nerve strands flanking the medio-frontal visceral nerve. One to two medio-lateral visceral nerves lie adjacent to the medio-frontal nerve, which adjoins one to three medio-lateral visceral nerves. The two latero-visceral nerves, however, are bilaterally symmetric and can be found in every each individual (Figure 2d-f).

#### Tentacle sheath nerves

Each zooid possesses four main tentacle sheath nerves that arise from the cerebral ganglion. The number of nerves (or tentacle sheath nerve roots), however, differ between zooids, and the nerves are usually asymmetrical. In most cases two to three nerves fuse, after the emergence from the ganglion, into a single neurite bundle. In particular, at the proximal side, close to the ganglion, some interconnections between the nerves are apparent. However, no specific pattern was found in the number of these interconnections and other ramifications (Figure 2d, e). The four tentacle nerves project through the tentacle sheath towards the aperture of the zooid from where they continue into the body wall/cystid. Within the cystid hardly any nerves are stained (Figure 2d-f).

## Discussion

### Tentacle innervation

With the exception of the Phylactolaemata [4], most adult ectoprocts have four to six tentacle nerves. In ultrastructural investigations of the tentacles four basiepidermal tentacle nerves per tentacle were found in the cheilostome *Electra pilosa* [23] and the ctenostome *Flustrellidra hispida* [37]. In the cheilostome *Cryptosula pallasiana* two additional basiperitoneal tentacle nerves were detected [18]. Less information about the tentacle innervation is available for the Stenolaemata. In the cyclostome *Crisia eburnea* (Stenolaemata) also four tentacle nerves were reported [38]. Accordingly, mainly four tentacle nerves per tentacle appear to be present in ectoprocts. These usually comprise one abfrontal nerve, one medio-frontal nerve and paired latero-frontal nerves. The position of the four tentacle nerves is usually described as basiepidermal. Only *Flustrellidra hispida* (Ctenostomata), *Membranipora membranacea* (Cheilostomata) [22], *Farella repens* (Ctenostomata) and *Alcyonidium* sp. (Ctenostomata) [31] and *Cryptosula pallasiana* (Cheilostomata) [18] seem to possess two basiperitoneal (motoric) and two basiepidermal (sensory) tentacle nerves. As already mentioned, *E. pilosa* (Cheilostomata), which is closely related to *M. membranacea* (both Malacostega), also possesses four basiepidermal tentacle nerves per tentacle. In *E. pilosa* the latero-frontal nerves were found to have an intertentacular origin. They branch off from the circum-oral nerve ring and bifurcate via an intertentacular fork from where one nerve projects into a tentacle. The abfrontal and medio-frontal nerves branch off directly from the circum-oral nerve ring [23]. In adult *Hislopia malayensis* (Ctenostomata) four basiepidermal tentacle nerves were found. The abfrontal and latero-frontal tentacle nerves have intertentacular origins, whereas the medio-frontal tentacle nerves branch off directly from the circum-oral nerve ring in *H. malayensis* [32]. The situation in *Paludicella articulata* (present study), which also possesses four basiepidermal tentacle nerves, mostly resembles the situation found in *H. malayensis*. In both species three tentacle nerves have intertentacular origins, with the medio-frontal tentacle nerves directly emanating from the circum-oral nerve ring (i.e., the cerebral ganglion). The abfrontal nerve origin in *P. articulata* represents also a point of amalgamation with nerves arising from the intertentacular fork similar to the conspicuous abfrontal nerve bodies in *H. malayensis* from where also a single abfrontal nerve extends [32].

Until now *Cryptosula pallasiana* (cheilostome) remains the only species for which six tentacle nerves have been described [18]. The presence of the additional two basiperitoneal tentacle nerves does not apply to *P. articulata* in the current study. Instead of two basiperitoneal tentacle nerve strands, basiperitoneal cells were found. These cells include numerous microtubules, but no bundles of neurotubules or nerve fibres were found. Therefore, and because they never appear as distinct fibers or strands, it appears likely that they constitute basiperitoneal cells instead of nerves. These cells have been described in virtually all ultrastructural investigations: in the cheilostome *E. pilosa* [23] and the phylactolaemates *Fredericella sultana*, *Plumatella emarginata*, *Lophopus crystallinus* [17] and *Asajirella gelatinosa* [4].

### The structure of the cerebral ganglion

The nervous system of *Paludicella articulata* essentially consists of a cerebral ganglion, a circumoral nerve ring, tentacle innervation, tentacle sheath nerves and visceral innervation. The main part of the nervous system is restricted to the basis of the lophophore. This is in line with all other ectoprocts investigated, which also possess a circum-oral or circum-pharyngeal nerve ring. In all ectoproct species investigated so far the central nervous system originates from an invagination of the prospective anal side of the pharyngeal epithelium during the budding process. Therefore, a lumen within the cerebral ganglion in the early developmental stages appears [29,30,39]. This has also been described for *P. articulata* [35]. Until now, the prevailing opinion was that the only ectoproct group which as adults still bears a lumen within their cerebral ganglion is the Phylactolaemata [4]. Several studies have described a vesicle-like cerebral ganglion in adult Phylactolaemata [14,20,21,30,39]. The first ctenostome species where a lumen was found is *P. articulata* (current study). The phylactolaemates *Fredericella sultana* and *Plumatella emarginata* also bear a fluid-filled cavity within their cerebral ganglion. Furthermore, a neuroepithelial organization of the cells lining the lumen with apical adherens junctions is present in these two species [27]. In the ctenostome *P. articulata* (present study) a neuroepithelial organization of the ganglion cells was also found. These interconnections of the nerve cells are also predominately located around the lumen. However, in *H. malayensis*, another ctenostome representative, the ganglion never contains a lumen but is compact from late budding stages onwards [32].

In the phylactolaemates *Lophopus crystallinus* [22], *F. sultana* and *P. emarginata* [27], as well as in the ctenostome *P. articulata* (current study), the perikarya of the cerebral ganglion face the lumen. In the cerebral ganglion of *H. malayensis* one conspicuous prominent perikaryon was described based on histological sections. It is situated centrally within the ganglion and is larger than the other nerve cells [32]. In the cerebral ganglion of *P. articulata* all cells appear morphologically similar. No difference concerning the abundance and the distribution of cell organelles within these ganglionic cells were found. This is in contrast to the ctenostome *H. malayensis* [32]. In the phylactolametes *F. sultana* and *P. emarginata* additional neurosecretory cells are present which appear to be missing in the Ctenostomata [27]. In the ctenostome *Flustrellidra hispida* two different types of ganglion cells were described histologically, which differ in size [22]. In the cheilostome *E. pilosa* three different categories of neurons were found by ultrastructural studies. These are neurons with characteristic neurotubules, secretory and glial cells [24]. In ultrastructural investigations of the phylactolaemates *F. sultana* and *P. emarginata* [27] no difference between the ganglionic cells were found, which most likely resembles the situation found in *P. articulata* (current study).

### Visceral innervation

The visceral innervation has been analyzed in several ectoproct species [21,22,24]. In malacostegine cheilostomes ectoprocts two pairs of visceral nerves were described. A thick median visceral nerve is reported to project to the esophagus and lateral visceral nerves emerge from a separate visceral ganglion and project from the apex of the esophagus to the anus. These visceral nerves are connected via annular ramifications between the subdivisions of the gut [24]. *Electra pilosa*, *E. verticilliata* and *Membranipora membranacea* (Cheilostomata) also possess a prominent medio-visceral nerve, two medio-lateral visceral nerves and two lateral visceral nerves [26]. Only one major nerve is described to descend at the frontal side of the pharynx in *Cryptosula pallasiana* [18]. In *Zoobotryon verticillatum* (Ctenostomata) a frontal and lateral esophageal nerve were detected [21]. In *Flustrellidra hispida* the situation is similar, with one median visceral nerve and two fine medio-lateral nerves which are interconnected via a commissure and an asymmetrical tripolar nerve cell [22]. Most visceral nerves arise either from the cerebral ganglion or from a visceral ganglion, a small accessory ganglion located proximally of the cerebral ganglion. In *P. articulata* all nerves emerge from the cerebral ganglion and a comparable, prominent medio-visceral nerve was also detected. The three to five medio-lateral visceral nerves were found to be asymmetrically arranged. However, the two lateral visceral nerves appear bilaterally symmetrical. Taken together, the visceral innervation of *P. articulata* most closely resembles that of the cheilostomes with its three types of visceral nerves [26], all emerging from the cerebral ganglion.

### Tentacle sheath nerves

In the phylactolaemate *Cristatella mucedo,* two pairs of prominent tentacle sheath nerves – frontal and basal - that arise from the cerebral ganglion were described [21]. In *Flustrellidra hispida* (Ctenostomata) two tentacle sheath nerves arise from the cerebral ganglion and bifurcate to form four distinct tentacle sheath nerves. The distal pair of the tentacle sheath nerves projects towards the aperture and finally ends as distal plexus. The other two nerves surround the attachment of the tentacle sheath [22]. In the current investigation, the tentacle sheath nerves were detectable among the main nerves that branch off the cerebral ganglion. The roots of the tentacle sheath nerves always fuse into four distinct nerves. However, the tentacle nerve roots that arise from the cerebral ganglion can differ in number and appear asymmetrically positioned. In *E. pilosa* (Cheilostomata) also two pairs of main tentacle sheath nerves emerge from the cerebral ganglion. The more laterally situated and three-branched nerves, called “trifid nerves”, are thin bundles that merge at the aperture [31]. These trifid nerves bend towards the esophagus before they again bend in distal direction and project towards the tentacle sheath. The inner median “direct nerves” are thick strands without branches until they merge with the axial branches of the trifid nerves. The axial branches of the trifid nerve extend distally, almost parallel to the “direct nerves”. The merged neurite bundle finally form the compound tentacle sheath nerves [23]. A paired parietal plexus or “cystidial nerves”, (cf. [25]) is situated parallel to the compound tentacle sheath nerves in the cystid. The parietal plexus is also connected to the cerebral ganglion via the tentacle sheath [24,40]. The tentacle sheath innervation of *P. articulata* most closely resembles that of cheilostomes [23]. One pair of “direct nerves” with their characteristic bending in distal and afterwards again in apical direction appears also in the ctenostome *P. articulata*. The so called “trifid nerves”, which merge with the direct nerves, are present in a similar pattern. The triple-branching of these nerves is not clearly recognizable. The merged “direct” and comparable “trifid” nerve appears to be similar to the great tentacle sheath nerve [4]. As mentioned before, the number of all tentacle sheath nerves arising from the cerebral ganglion is odd and the nerves are arranged asymmetrically. An asymmetrical arrangement of nerves has so far not been described for any other ectoproct species.

## Conclusions

The anatomy of the adult ectoproct nervous system, which is concentrated at the lophophoral base, has been subject of several investigations in the Gymnolaemata and Phylactolaemata. However, the nervous system of the Stenolaemata remains almost unstudied. Based on the available data, a trend is recognizable concerning the tentacle innervation from intertentacular origins in the basal Phylactolaemata towards direct innervation from the cerebral ganglion in Gymnolaemata (cf. [31]). The state in the paraphyletic Ctenostomata shows three out of four nerves to originate from the ganglion [32], whereas the Cheilostomata have only two of the four [23]. The current study supports the situation of the tentacle innervation in the Ctenostomata as observed in the *Hislopia malayensis* [32]. In order to verify this trend, additional cheilostome and cyclostome species need to be studied.

The lumen within the cerebral ganglion in shape of a small vesicle is found in all ectoprocts during ontogeny. The differentiation of the adult ganglion seems to lead to the obliteration of the ganglion lumen in most ectoprocts (cf. [32]), whereas its persistence into the adult is so far only documented for the Phylactolaemata [20,27]. The present study is the first to assess a lumen in the adult of *P. articulata*, a non-phylactolaemate ectoproct. Owing to its small size, it is quite possible that this lumen is more widespread and persistent than previously thought. It seems that *P. articulata* has retained the lumen like the basal Phylactolaemata, or, instead, has independently evolved in the adult condition. For a proper evaluation, however, additional species need to be investigated.
